# The relationship between social support and erectile dysfunction in middle-aged and older males

**DOI:** 10.3389/fpubh.2024.1332561

**Published:** 2024-05-09

**Authors:** Danqing Hu, Yan Zhang, Yue Zhang, Yu Liu, Jing Han

**Affiliations:** ^1^School of Nursing, Qingdao University, Qingdao, Shandong, China; ^2^Department of Critical Care Medicine, The Affiliated Hospital of Qingdao University, Qingdao, Shandong, China; ^3^School of Nursing, Hong Kong Polytechnic University, Hong Kong, China; ^4^Department of Women's Health Care, Qingdao Maternal & Child Health and Family Planning Service Center, Qingdao, Shandong, China

**Keywords:** erectile dysfunction, middle-aged, older adult, sexual health, social support

## Abstract

**Background:**

Erectile dysfunction (ED) is a prevalent condition that affects middle-aged and older men, impacting their sexual health and overall wellbeing. We aimed to investigate the relationship between social support and ED among this specific population.

**Methods:**

Data were collected from the National Health and Nutrition Examination Survey. Social support was assessed through various dimensions, including emotional support, material support, and network support. Multivariate logistic regression was performed to examine the association between social support and ED, and a propensity-score-matched (PSM) analysis was further conducted.

**Results:**

Among 1938 middle-aged and older males in the United States, 49.9% had a history of ED. ED was more prevalent in older individuals and those with comorbidities such as hypertension, prostate disease, higher serum creatinine level, and mental problems. Males with lower social support scores had a higher weighted rate of ED (*P* < 0.001). After adjusting for multiple variables in logistic regression analysis, a higher social support score was associated with a 19% lower likelihood of ED (weighted odds ratio [OR] 0.81, 95% confidence interval [CI] 0.66–0.98, *P* = 0.032). The association remained consistent after propensity score matching (OR 0.80, 95% CI 0.66–0.98, *P* = 0.028).

**Conclusion:**

Social support appears to be associated with a reduced risk of ED in middle-aged and older men. Further research is needed to better understand this relationship and explore interventions that enhance social support, potentially leading to improved sexual health outcomes.

## Introduction

Erectile dysfunction (ED) is a common male sexual dysfunction characterized by the inability to achieve or maintain an erection sufficient for satisfactory sexual intercourse (1, 2). Erection is caused by the equilibrium of blood flow into and out of the penis. The process of achieving and maintaining an erection involves a complex interplay of psychological, neural, and vascular pathways, collectively contributing to the physiological response of the penile vascular system (3–6). Conditions that cause alternations in the blood flow of the penis are strongly associated with common issues such as hypertension, obesity, and smoking (7–10). Apart from the conventional causes mentioned earlier, there is growing recognition of the role of social factors in the development and management of ED (11).

Social support encompasses various forms of assistance, including emotional support, informational support, and tangible help, that individuals receive from their social networks (12). It plays a vital role in promoting both mental and physical wellbeing (13). The resources, assistance, and emotional comfort provided by social support may be associated with protection against the psychological and physiological processes of ED (14). Understanding the relationship between social support and ED is essential for identifying factors that affect sexual health outcomes. Moreover, it is crucial to recognize that social support is a modifiable factor. By identifying the role of social support associated with ED, we can pave the way for developing interventions aimed at enhancing social support and potentially improving sexual health. Previous research suggests that psychological support may have a significant impact on young men with ED (11). However, the relationship between social support and ED is uncertain in middle-aged and older male groups.

Therefore, we conducted an investigation to examine the relationship between social support and ED among middle-aged and older males in the United States, utilizing nationally representative data from the National Health and Nutrition Examination Survey (NHANES). Our study examined various forms of social support, including emotional support, material support, and network support, to determine their impact on the risk of ED, aiming to provide a comprehensive understanding of the associations between different aspects of social support and ED among middle-aged and older men.

## Materials and methods

### Study design and population

**Figure 1:**
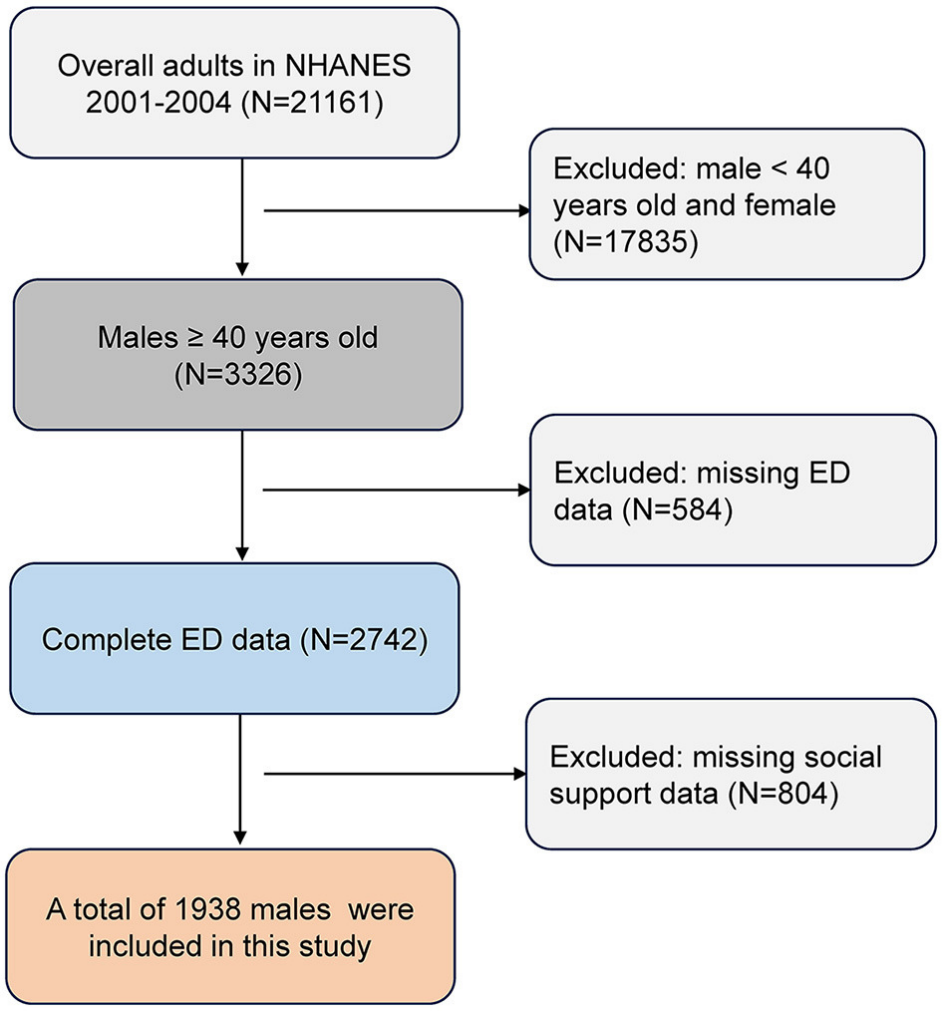
Flow chart of the screening process for the selection of eligible participants. ED, erectile dysfunction; NHANES, National Health and Nutrition Examination Survey.

The NHANES is a nationwide survey conducted by the Centers for Disease Control and Prevention in order to provide health and nutrition data of United States population. Detailed information about the design and methodology of the survey is available in the NHANES website (https://www.cdc.gov/nchs/nhanes/index.htm). In brief, the study adopts a stratified, multi-stage probabilistic sampling design of the civilian, non-institutionalized population in the USA. Participants are initially interviewed at home and subsequently undergo a physical examination at a mobile screening center. The survey has received approval from the National Center for Health Statistics Research Ethics Review Board, and written consents are obtained from participants prior to the interview and examination phases. For data users and researchers throughout the world, survey data are available on the internet. For our study, we utilized NHANES data collected between 2001 and 2004. Data on ED was available for males aged 20 years and older. Our cross-sectional analysis focused on a cohort of 1,938 males aged 40 years and older, for whom complete data on both ED and social support were available. Please refer to Figure 1 for a flowchart illustrating subject inclusion and exclusion criteria.

### Study variables

Social support comprised three components: emotional support, material support, and network support (number of close friends). To assess emotional support, participants were asked the following question: “Can you count on anyone to provide you with emotional support such as talking over problems or helping you make a difficult decision?” The response options were yes or no. For measuring material support, the question included was: “If you need some extra help financially, could you count on anyone to assist you, for example, by paying bills, housing costs, hospital visits, or providing you with food or clothing?” The response options were yes or no. The number of close friends was defined as “relatives or non-relatives with whom you feel comfortable, can discuss personal matters, and can rely on for help.” The population mean for this variable was 5 friends; thus, it was categorized as having ≥5 friends and < 5 friends, following the literature (15). These questions were selected from the Yale Health and Aging Study and the Social Network Index - Alameda County Study (16, 17). The sum of the social support scores ranged from 0 to 3.

ED outcomes were determined based on the self-reported single-item questionnaire from the Massachusetts Male Aging Study, which inquired about the ability to achieve and maintain an erection adequate for satisfactory intercourse. The available response options were: “always or almost always able,” “usually able,” “sometimes able,” and “never able.” We defined ED as individuals who reported being “sometimes able” or “never able,” consistent with previous studies (18, 19).

Other information included demographic characteristics (age, marital status, household income, body mass index, and waist circumference), lifestyle characteristics (smoking, abnormal alcohol intake, and physical activity), and medical characteristics (hypertension, prostate disease, triglyceride-glucose index, serum creatinine, mental problems, and regular prescription medications). Smoking was defined as having smoked at least 100 cigarettes in life. Abnormal alcohol intake was defined as consuming at least one drink per day. Participants completed a physical activity questionnaire to self-report their average level of physical activity each day. Activity levels were classified as follows: “none” for individuals who reported sitting most of the day without much walking, “light” for those who reported standing or walking a lot but without much lifting or carrying, “Moderate” for those reported often lifting light loads or climbing stairs/hills, and “Vigorous” for those engaged in heavy work or carrying heavy loads, as indicated in the questionnaire. Hypertension was defined as systolic blood pressure ≥140 mmHg, diastolic blood pressure ≥90 mmHg, having a history of hypertension, or taking prescription for antihypertension medication. Mental problems were defined based on the question: “During the past 12 months, have you seen a mental health professional, such as a psychologist, psychiatrist, psychiatric nurse, or clinical social worker, about your health?” The response options were yes or no. The triglyceride-glucose index was calculated with this formula: Ln [fasting triglycerides (mg/dL) × glucose (mg/dL)/2] (20).

### Statistical analysis

Continuous variables were described using either mean (standard deviation, SD) or median (interquartile range, IQR), and comparisons between groups were conducted using unpaired *t*-test or Mann-Whitney *U*-test, depending on the data distribution. Categorical variables were presented as frequencies and percentages, and group comparisons were performed using the chi-squared test. The association between social support and ED was assessed using logistic regression models. Multivariable analyses adjusted for confounding factors, including all variables listed in Table 1.

**Table 1:** Comparison the characteristics of participants with or without erectile dysfunction.

	**Entire sample**		***P* value**	**PSM sample**		***P* value**
	**Not ED (*****n** =* **971)**	**ED (*****n** =* **967)**		**Not ED (*****n** =* **312)**	**ED (*****n** =* **312)**	
**Demographic characteristics**						
Age (years)	54.0 (10.8)	68.0 (10.6)	< 0.001	65.6 (10.0)	64.9 (9.8)	0.404
Household income			< 0.001			0.849
< $20,000	182 (11.2)	275 (21.2)		72 (23.1)	70 (22.4)	
≥$20,000	740 (88.8)	623 (78.8)		240 (76.9)	242 (77.6)	
Marital status			< 0.001			1.000
Married or living with a partner	752 (81.1)	727 (79.5)		245 (78.5)	245 (78.5)	
Living alone	218 (18.9)	240 (20.5)		67 (21.5)	67 (21.5)	
Body mass index (kg/m^2^)	28.3 (4.5)	28.8 (5.5)	0.195	28.5 (4.5)	28.6 (5.4)	0.765
Waist circumference (cm)	102.7 (12.0)	106.1 (14.5)	0.001	104.3 (12.1)	104.5 (14.2)	0.836
**Lifestyle characteristics**						
Smoking	612 (61.9)	685 (70.9)	< 0.001	220 (70.5)	218 (69.9)	0.861
Abnormal alcohol intake	83 (9.3)	87 (14.2)	< 0.001	42 (13.5)	42 (13.5)	1.000
Physical activity			< 0.001			0.814
None	210 (20.9)	330 (32.2)		71 (22.8)	81 (26.0)	
Light	510 (51.2)	496 (50.7)		175 (56.1)	169 (54.2)	
Moderate	163 (18.9)	108 (13.3)		51 (16.3)	49 (15.7)	
Vigorous	85 (9.0)	31 (3.7)		15 (4.8)	13 (4.2)	
**Medical characteristics**						
Hypertension	473 (43.9)	666 (66.5)	< 0.001	201 (64.4)	191 (61.2)	0.407
Prostate disease	164 (14.4)	361 (35.4)	< 0.001	82 (26.3)	86 (27.6)	0.718
Triglyceride-glucose index	8.8 (0.02)	8.8 (0.04)	0.043	8.8 (0.6)	8.8 (0.7)	0.688
Serum creatinine (mg/dL)	1.0 (0.6)	1.1 (0.7)	< 0.001	1.0 (0.2)	1.1 (0.6)	0.381
Mental problems	49 (6.1)	55 (6.9)	< 0.001	21 (6.7)	19 (6.1)	0.744
Prescription medications	575 (59.1)	815 (84.7)	< 0.001	232 (74.4)	235 (75.3)	0.782

Data of entire sample are presented as weighted mean and standard deviation (SD), or number of participants and weighted percentage. Data of PSM sample are presented as mean and standard deviation (SD), or number of participants and percentage. ED, erectile dysfunction; PSM, propensity-score-matched.

Sensitivity analysis of propensity score matched (PSM) analysis was conducted to further control for confounding, matching cases in a 1:1 ratio with a caliper size of 0.02. The propensity score (PS) was calculated in a logistic regression model that included the covariates listed in Table 1. Group differences were evaluated using standardized mean differences (SMD). With SMD < 10.0% indicating relatively small imbalances between the groups (21).

Subgroup analyses were conducted to explore the relationship between social support and ED within different groups. These analyses involved examining interaction effects, which assess whether the association between social support and ED varies significantly across subgroups. The Wald test was employed to calculate interaction *p*-values, adjusting for other variables alongside grouping factors. Significant interactions were interpreted by assessing the impact of social support on ED within each subgroup, facilitating the detection of meaningful variations across groups.

Appropriate sampling weights were taken into account to ensure a nationally representative estimate in this study. R (version 3.4.2) and Stata 14.0 (Stata, College Station, TX, USA) were used for data analyses. Two-sided *P* values < 0.05 were considered statistically significant.

## Results

### Patients' characteristics

This study involved a total of 1,938 males, among which 967 (49.9%) reported a history of ED. The characteristics of the study population are presented in Table 1. Men without ED were younger (54.0 years vs. 68.0 years) and had a slightly lower waist circumference (102.7 cm vs. 106.1 cm), and serum creatinine level (1.0 mg/dL vs. 1.1 mg/dL). Yet, men with ED had a higher weighted prevalence of hypertension (66.5% vs. 43.9%), prostate disease (35.4% vs. 14.4%), and mental problems (6.9% vs. 6.1%) than those without ED (all *P* < 0.05). Detailed data on the characteristics of the study population are presented in Table 1.

### Association between social support and ED

**Figure 2:**
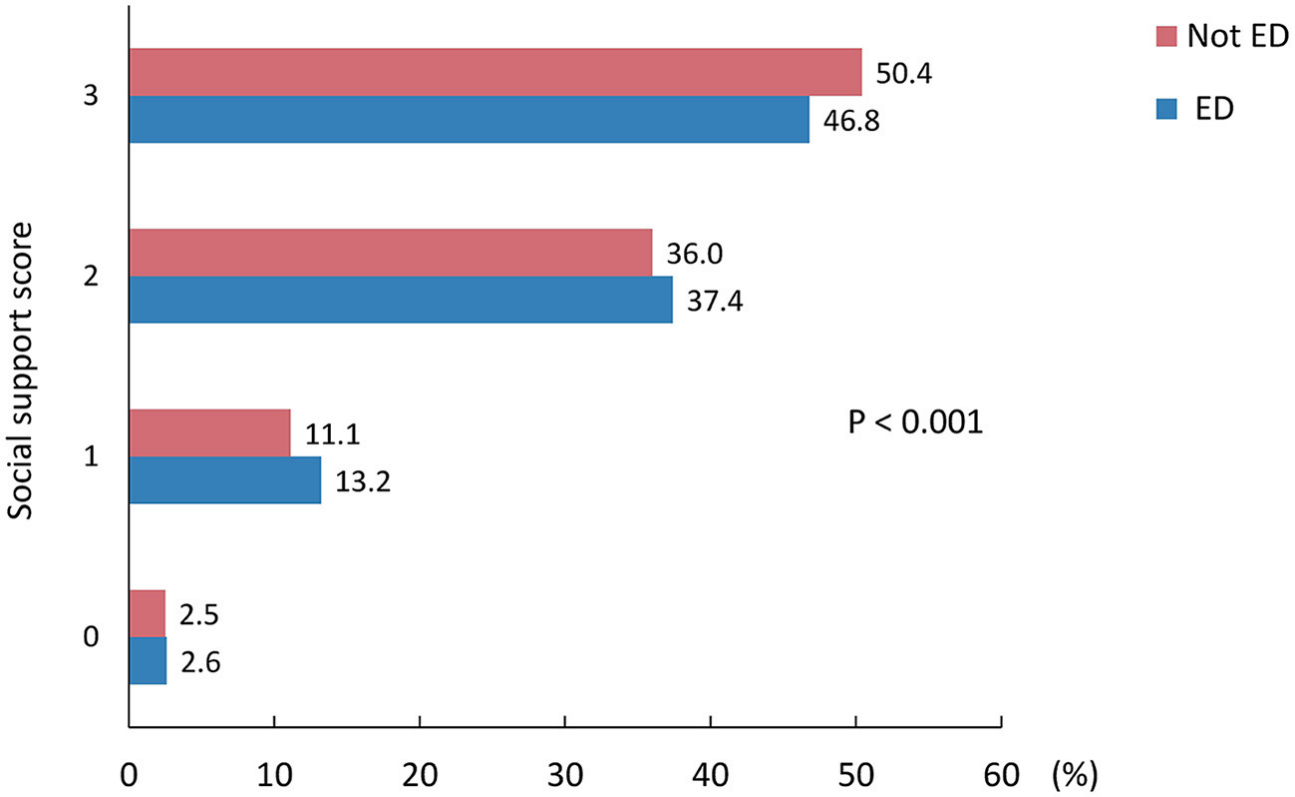
The weighted rate of erectile dysfunction in middle-aged and older males with different social support scores. ED, erectile dysfunction. The red bar represents the weighted rate of males without erectile dysfunction, while the blue bar represents the weighted rate of males with erectile dysfunction.

Males with higher social support scores demonstrated lower rates of ED (*P* < 0.001), as shown in Figure 2. In the univariate logistic regression analysis, there was no significant association between the social support score and ED (weighted odds ratio [OR], 0.91; 95% confidence interval [CI], 0.80–1.03; *P* = 0.137). However, after adjusting for age in model 1 (weighted OR, 0.82; 95% CI, 0.71–0.96; *P* = 0.016) and performing multivariable adjustments in model 2 (weighted OR, 0.81; 95% CI, 0.66–0.98; *P* = 0.032), significant inverse associations between social support and ED were observed. Similar results were observed in the unweighted sample (Table 2).

**Table 2:** The association between social support and erectile dysfunction among middle-aged and older males.

	**Unadjusted**		**Model 1**		**Model 2**	
	**OR (95%CI)**	***P*** **value**	**OR (95%CI)**	***P*** **value**	**OR (95%CI)**	***P*** **value**
Weighted	0.91 (0.80–1.03)	0.137	0.82 (0.71–0.96)	0.016	0.81 (0.66–0.98)	0.032
Unweighted	0.91 (0.81–1.01)	0.079	0.83 (0.73–0.95)	0.005	0.80 (0.68–0.96)	0.014
Propensity-score-matched	0.80 (0.66–0.98)	0.028	/		/	

Model 1 adjusted for age; Model 2 adjusted for age, household income, marital status, body mass index, waist circumference, smoking, abnormal alcohol intake, physical activity, hypertension, prostate disease, triglyceride-glucose index, serum creatinine, mental problems, and prescription medications. CI, confidence interval; OR, odds ratio.

For different types of social support, no significant association was observed between emotion support (weighted OR, 0.74; 95%CI, 0.27–2.04; *P* = 0.554), material support (weighted OR, 0.71; 95%CI, 0.47–1.06; *P* = 0.092), and network support (weighted OR, 0.82; 95%CI, 0.60–1.10; *P* = 0.177) and ED after multivariate adjustment in model 2 (Table 3).

**Table 3:** The association between different types of social support and erectile dysfunction among middle-aged and older males.

	**Unadjusted**		**Model 1**		**Model 2**	
	**OR (95%CI)**	***P*** **value**	**OR (95%CI)**	***P*** **value**	**OR (95%CI)**	***P*** **value**
Emotional support	0.95 (0.52–1.75)	0.876	1.04 (0.45–2.40)	0.928	0.74 (0.27–2.04)	0.554
Material support	0.73 (0.57–0.92)	0.010	0.73 (0.56–0.97)	0.029	0.71 (0.47–1.06)	0.092
Network support	1.00 (0.78–1.28)	0.983	0.77 (0.60–1.00)	0.053	0.82 (0.60–1.10)	0.177

Weighted OR and 95%CI. Model 1 adjusted for age; Model 2 adjusted for age, household income, marital status, body mass index, waist circumference, smoking, abnormal alcohol intake, physical activity, hypertension, prostate disease, triglyceride-glucose index, serum creatinine, mental problems, and prescription medications. CI, confidence interval; OR, odds ratio.

### Sensitivity analysis

The PSM analysis was performed to further confirm the association between social support and ED. After PSM, 312 males with ED were matched with 312 males without ED (Table 1). The SMD for most covariates were lower than 10%, indicating that PS for the two groups significantly overlapped (see Supplementary Figure 1). The characteristics between these two groups were re-compared, and all of the characteristics were comparable (*P* < 0.05) (Table 1). The independent association between social support and ED was also found in the PSM sample (OR, 0.80; 95%CI, 0.66–0.98; *P* = 0.028) (Table 2).

### Subgroup analyses

The associations between social support and the risk of ED were assessed through subgroup analyses based on various characteristics, including age (< 65 years or ≥65 years), household income (< $20,000 or ≥$20,000), marital status (living alone or married/living with a partner), body mass index (< 30 kg/m^2^ or ≥30 kg/m^2^), smoking (no or yes), abnormal alcohol intake (no or yes), hypertension (no or yes), prostate disease (no or yes), mental problems (no or yes), and prescription medications (no or yes). No statistically significant interactions were found between these variables and social support in relation to ED (all *P* for interaction > 0.05) (see Supplementary Figure 2).

## Discussion

Based on a nationally representative cross-sectional study of United States adults, we investigated the association between social support and the risk of ED in middle-aged and older men. In this study, nearly half of these middle-aged and older males experienced ED. Males with higher social support scores had a lower rate of ED. After adjusting for confounding factors, higher social support was significantly associated with a reduced risk of ED.

The prevalence of ED in our study was approximately 50%, which aligns with data reported in the Massachusetts Male Aging Study, where 52% of males aged 40 to 70 years old experienced some degree of ED (22). ED has a profound impact on the quality of life for both individuals experiencing it and their partners, often leading to significant relationships challenges. Previous study demonstrated a strong association between couple's relationship deterioration and ED (23). However, there is limited research on the influence of social relationships on ED. The results of our study showed that males with higher social support scores demonstrated lower rates of ED, consistent with a recent study conducted in China found that men with ED had significantly lower social support scores compared to those without ED, suggesting a potential bidirectional relationship between ED and social relationships (24). In contrast to previous studies that have primarily emphasized the importance of psycho-social support for young men with ED (6, 11), our study revealed a significant negative correlation between social support and ED in middle-aged and older males, even after controlling for confounding variables.

In our study, we found that while single social support items were not significant, the sum score yielded significant results. We suggest that this may be due to the cumulative effect of multiple sources of support being more influential than considering individual dimensions separately. We believe that middle-aged and older men at risk of ED may benefit from comprehensive social support, including emotional, material, and network support. Achieving this requires collaboration among government agencies, healthcare professionals, communities, families, and individuals.

Social support is a crucial resource that serves as a protective buffer, effectively counteracting the detrimental effects of health conditions (25). Its impact becomes particularly significant in middle-aged and older individuals, where the positive effects of social support are more pronounced. Research indicates that the structure and dynamics of social support networks undergo change as people age (26). Specifically, older adults tend to describe networks comprised of smaller, closely-knit groups, which contrasts with the larger networks commonly found among younger individuals (27). Social support can provide psychological comfort and assistance, enabling men to effectively manage stress and anxiety related to their sexuality, thereby promoting positive mental wellbeing and mitigating the adverse effects of sexual stress on erectile function (28). Moreover, maintaining a healthy lifestyle has been consistently linked to a lower risk of ED (29, 30). Healthy lifestyle choices are not just an individual choice, but can also be influenced by social relationships. The companionship and advice from family and close friends contribute to the development of healthy habits, the sustenance of stable relationships, and ultimately a reduced risk of ED. Additionally, various types of social support can act as a source of motivation and accountability for men to prioritize their health. It was reported that if experiencing ED, more than one-fifth of individuals turned to their friends for help (31). Through such network support, individuals are encouraged to pursue regular medical check-ups, adhere to prescribed treatments, and proactively seek professional help whenever required.

Our results have several important implications. Firstly, the significant inverse association between social support and ED among middle-aged and older men highlights the importance of addressing social support in sexual health interventions and policies. Governments and health agencies should consider incorporating social support components into sexual health programs, especially for middle-aged and older men. Secondly, acknowledging the potential impact of social factors on the development and management of ED, it is crucial for healthcare practitioners to place greater emphasis on assessing and addressing social support needs when evaluating and treating individuals with ED. Incorporating assessments of social support into routine clinical practice not only facilitates the identification of patients at higher risk but also allows for the customization of interventions based on individual needs, thus enhancing the clinical relevance of treatment strategies. Healthcare providers can work collaboratively with social workers, counselors, and community support organizations to provide comprehensive care that addresses both the physical and psychosocial aspects of ED. In addition to its relevance in the context of ED, it is noteworthy that social support has been extensively linked to improved mental and physical health outcomes within this age cohort, emphasizing the broader benefits of fostering strong social connections and networks (32, 33).

To the best of our knowledge, this study is the first to investigate the association between social support and ED in middle-aged and older men. However, there are certain limitations to be acknowledged. The main limitation of this study is the use of a self-reported questionnaire method to assess ED, which may not accurately reflect the true prevalence of ED. However, the validity of the measurement in our study has been previously tested, and the prevalence of ED in our study was similar to previous reports, which increases our confidence in the results (22, 34, 35). We posit that self-reported experiences of ED may be more relevant for perceived social support than objectively assessed ED. Nonetheless, the data cannot be generalized to men whose ED has been objectively established. It's important to acknowledge that one limitation of our study is the lack of more recent ED data in the NHANES database. However, the prevalence of ED in the NHANES database from 2001–2004 is comparable to previous and recent ED prevalence data reported in studies (22, 36). Despite the age of the data, it remains valuable for examining trends and associations related to ED and social support. Additionally, the data are based on single items and binary response options, making them crude measures that may overlook possible nuances. Furthermore, the cross-sectional design of the study limits our ability to establish a causal association between social support and ED, given the potential for reverse causality or bidirectionality. Therefore, further prospective studies are essential to clarify the role of social support in preventing ED. Furthermore, despite our efforts to adjust for potential confounders, it's important to acknowledge the possibility of residual confounding. To address this concern, we conducted PSM analyses to further reduce bias. However, conducting multiple hypothesis tests may potentially elevate the risk of Type I errors. It's worth noting that the absence of explicit multiple testing correction methods in our data analysis represents a limitation. Before integrating the results into clinical practice, confirming their clinical relevance is crucial. However, in our study, although statistical significance was prioritized, it's important to note that achieving statistical significance is more likely with larger sample sizes, but the clinical significance may not always align. We must acknowledge the limitation of not directly assessing clinical significance through effect sizes, which may necessitate further validation from additional studies.

## Conclusion

In conclusion, our study found a significant inverse association between social support and ED among middle-aged and older men in the United States. While our findings suggest a potential protective role of social support in preventing ED. Further prospective studies are needed to validate this hypothesis and elucidate the underlying mechanisms.

## Data availability statement

The original contributions presented in the study are included in the article/Supplementary material, further inquiries can be directed to the corresponding author.

## Ethics statement

The studies involving humans were approved by the National Center for Health Statistics Research Ethics Review Board. The studies were conducted in accordance with the local legislation and institutional requirements. Written informed consent for participation was not required from the participants or the participants' legal guardians/next of kin in accordance with the national legislation and institutional requirements.

## Author contributions

DH: Conceptualization, Data curation, Writing—original draft. YaZ: Data curation, Writing—review & editing. YuZ: Data curation, Writing—review & editing. YL: Data curation, Writing—review & editing. JH: Conceptualization, Methodology, Supervision, Writing—review & editing.
