# Supplementary material for: The relationship between social support and erectile dysfunction in middle-aged and older males
Source: Front Public Health. 2024 May 9;12:1332561. doi: 10.3389/fpubh.2024.1332561 (PMC11112009; doi:10.3389/fpubh.2024.1332561)
Supplement: Supplementary file 1 [file Data_Sheet_1.docx]

**Supplementary material**

**Supplementary Material Figure 1** Standardized mean differences of weighted, unweighted and propensity score-matched middle-aged and older males

**Supplementary Material Figure 2** Subgroup analyses of associations between social support and the risk of erectile dysfunction based on different characteristics


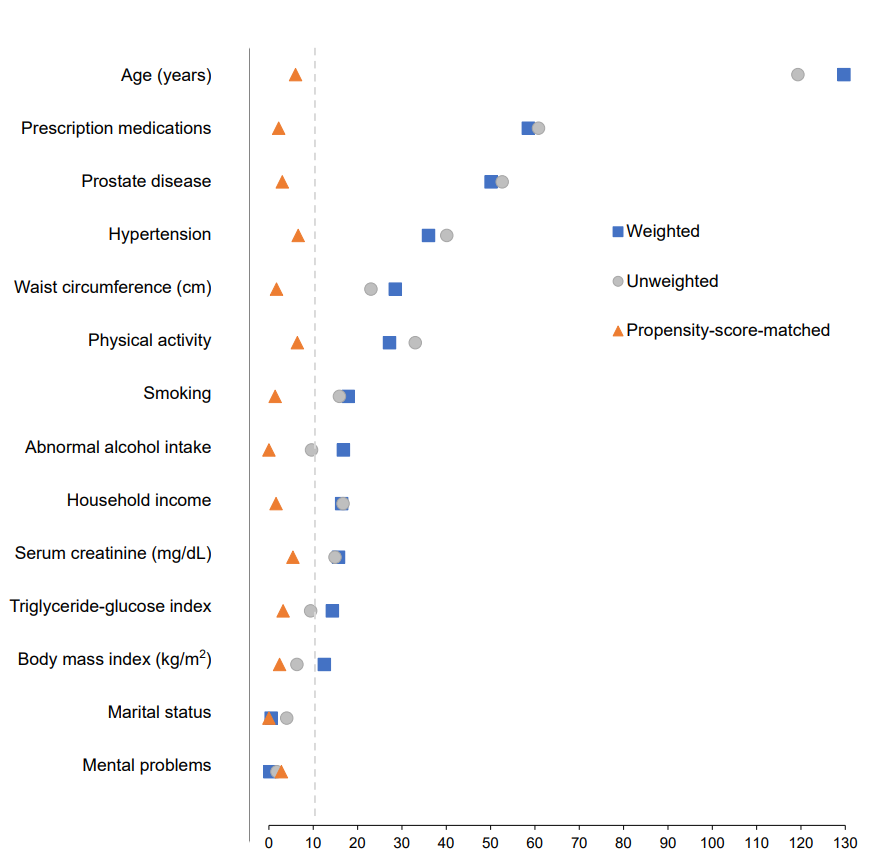


Supplementary Material Figure 1 Standardized mean differences of weighted, unweighted and propensity score-matched middle-aged and older males

Notes: Blue squares represent the Standardized Mean Difference (SMD) of the weighted sample, gray circles represent the SMD of the unweighted sample, and orange-red triangles represent the SMD of the sample after propensity score matching.


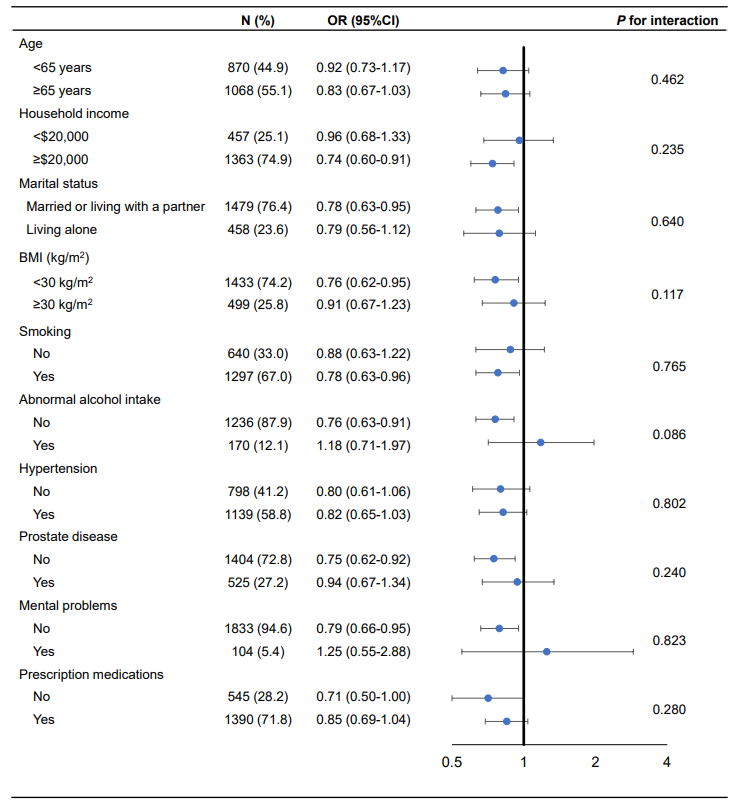


Supplementary Material Figure 2 Subgroup analyses of associations between social support and the risk of erectile dysfunction based on different characteristics
